# Experiments with Cundurango

**Published:** 1873-01-15

**Authors:** Edmund Andrews

**Affiliations:** Professor of Surgery in Chicago Medical College


### EXPERIMENTS WITH CUNDURANGO—ITS POWERFUL INFLUENCE ON ULCERS.—IT DOES NOT CURE CANCER.

BY EDMUND ANDREWS, A. M., M. D., PROFESSOR OF SURGERY IN CHICAGO MEDICAL COLLEGE.

The South American physicians chiefly praise cundurango for its power over ulcers, eruptions of the skin, and syphilis, but speak more cautiously of its value in cancer. In this country, on the contrary, surgeons seemed determined to use it only for the cure of cancer, and failing in that, to pronounce it a humbug.

Personal correspondence, however, with Dr. Chiribaga, Surgeon-in-Chief of the Army of Ecuador, and Dr. Casares, the clear-headed Professor of Surgery in Quito, satisfied me that these men had acquired their strong confidence in cundurango through an extended experience, and that consequently they were not likely to be wholly mistaken. The facts detailed by them, however, lacked fullness of statement, especially in the matter of diagnosis, so that I remained considerably at a loss to know in what the exact effect of the drug consisted, and to what forms of ulcers, etc., it was adapted.

To clear up this obscurity I undertook the subjoined experiments. Most of the patients were in Mercy Hospital, but a few were in the private practice of myself and others. The cundurango used was the fluid extract prepared by Bliss & Keene, of New York, and was administered exclusively by the stomach, none being applied locally except in one instance. The dose was a teaspoonful for adults, and proportionately for children, four times a day.

These experiments showed that, though cundurango has little or no influence over cancerous tumors, yet it possesses other qualities which render it one of the most remarkable substances ever introduced into the materia medica.

The first thing which strikes the surgeon experimenting with it, is its marvelous power to increase the growth of granulations in ulcers. Table I. will place the facts concisely before the reader. As the experiments are recent, many of the cases given in the tables are not complete, but only show their progress up to the present time.

What strikes one most forcibly in this table, is the almost invariable increase of granulations soon after the medicine was commenced. This change is visible in from two to ten days, and is sometimes very remarkable. The growth of granulations is followed by contraction of the ulcer, and often by rapid cicatrization. In some cases the remedy seemed after a time to lose its effect, but in none did it fail to bring at least some benefit. Most of the patients experienced an increase of the appetite, and many, but not all, a greater frequency of alvine discharges, so that constipation was often completely relieved by it. I did not observe any effect upon the kidneys, nor the other glands. The nervous system showed no evidence of being affected, except by a diminution of pain in certain cases. Some showed a decided increase of flesh, vigor and good spirits, as if elevated by the influence of a powerful tonic. This was best seen in one patient (case 7557, Table II.), who took double doses steadily for fifteen months.

The soft chancres and suppurating buboes were all cauterized with nitric acid at the outset, so that they should probably be considered as changed by the cautery from poisonous to ordinary ulcers. I have not tested the question of whether the remedy would succeed in an uncauterized soft chancre. On the whole, the indolent ulcers, following after the suppuration of buboes, did not as promptly respond to the medicine as ordinary ulcers, yet they were decidedly benefited.

In tertiary syphilis the results were excellent. This might have been foreseen, for the power of cundurango is most conspicuous in ulcerative diseases, and these are especially characteristic of tertiary syphilis. Even the eruptions of this stage, such as rupia, are ulcerative in their nature.

### TABLE I.

### CASES SHOWING THE EFFECTS OF CUNDURANGO IN COMMON ULCERS, IN SOFT CHANCRES AND IN THE ERUPTIONS AND ULCERATIONS OF TERTIARY SYPHILIS.

| NUMBER OF THE CASE IN MY RECORD. | AGE. | DISEASE, HISTORY, ETC. | CONDITION AT THE TIME OF COMMENCING THE MEDICINE. | SYMPTOMS AND PROGRESS WHILE TAKING THE REMEDY. |
|---|---|---|---|---|
| Case 7927 | 28 yrs. | A fibrous tumor was removed from each trochanter. Wounds became indolent ulcers, very inactive, and indisposed to heal. Took tincture of iron for a long time in vain. | Appetite poor, bowels costive, ulcers indolent, about one or two inches in diameter, and healing very slowly. | Commenced taking cundurango. In about four days the appetite improved, the bowels became regular, and the ulcers produced fresh granulations. They soon contracted rapidly in diameter, and in two weeks were nearly healed. There remain now only small unhealed points, which are again slow in their final closure. Dose, a teaspoonful four times a day of the fluid extract. |
| Case 7940 | 22 yrs. | Tertiary syphilis. Several indolent ulcers on each leg. Had iodide of potassium for a week with no benefit. | Ulcers indolent and unhealthy, with no tendency to heal. Discharge somewhat foul. | Commenced taking cundurango Oct. 26th in dram doses of fluid extract four times a day. Shortly began to improve, granulations sprouted, and all the ulcers but two healed by the 18th day. The remaining sores then became stationary. Finding a cessation of the improvement, the cundurango was discontinued and iodide of potassium again given for three weeks without any benefit. He was then placed again on cundurango, and in four days showed fresh granulations and renewed improvement. |
| Case 7942 | 55 yrs. | Probably tertiary syphilis. The patient first had a kind of indolent boil in various parts of the body, followed by a half scaly, half scabby eruption, of an illy defined character. He took iodide of potassium, tincture of iron, sulphuric acid, etc., at different times, without benefit. | But little appetite; bowels constipated; eruption persistent; several boils in various places. | Commenced taking cundurango Oct. 25th. Soon improved materially; appetite increased, constipation disappeared, eruption subsided and the boils ceased to present themselves. Took the remedy twenty-five days, and was discharged nearly cured. |
| Case 7963 | 33 yrs. | Tertiary syphilis. Has taken no medicine previously. | An eruption exists over the body, with scattered scabs resembling those of rupia, but rather small, accompanied with itching. | Commenced cundurango Nov. 29th. Improved rapidly; the itching ceased, the scabs fell off, and the patient was cured in eleven days. |
| Case 7079 | 3 yrs. | This child had a bad lapus on the forearm, nearly encircling it, which was cured by repeated operations and cauteries. Soon after the healing, the cicatrix ulcerated again. | The new ulcer is indolent, about an inch in diameter, and after being treated a week or two refuses to improve. Bowels constipated. | Commenced cundurango Nov. 11th. In twenty-four hours there was an appearance of fresh granulations, and in ninteen days the ulcer was cured. |
| Case 7151 | 22 yrs. | Soft chancres. Had a suppurating bubo in the groin, the contagion of which had planted a crop of ulcers all along the pubes. Cauterized the ulcers. | Ulcers feeble, unhealthy and having few granulations. Appetite imperfect. | Ordered cundurango. Appetite improved; bowels as before. In six days (the latest observation) the granulations were found increased in quantity. |
| Case 7115 | 24 yrs. | Soft chancres which have existed fifty-five days; also a suppurating bubo. | Two soft chancres and one suppurating bubo, all of which were cauterized after lancing the bubo. | Commenced cundurango. Appetite improved; bowels as before. In five days the chancres commenced contracting without perceptible increase of granulations. Now (9th day), the healing process is going on finely. |

TABLE I.—CONTINUED.

| NUMBER OF THE CASE IN MY RECORD. | AGE. | DISEASE, HISTORY, ETC. | CONDITION AT THE TIME OF COMMENCING THE MEDICINE. | SYMPTOMS AND PROGRESS WHILE TAKING THE REMEDY. |
|---|---|---|---|---|
| Case 7084 | 40 yrs. | Forearm and hand ripped up by the horn of a bull, from the fingers to the elbow. Considerable sloughing. When it was almost healed, a renewed ulceration attacked the cicatrix, and in three days nearly the whole scar was again an ulcer. | Ulcer increasing in size. No granulations at all. Cauterized edges two days with nit. acid. | Ordered cundurango. Appetite increased. By the fourth day the granulations were sprouting and the enlargement of the ulcer was arrested. By the sixth day, the granulation was still more vigorous, and the ulcer contracting. Seventeenth day, nearly healed. The remaining spot proving obstinate, the dose was doubled, when the healing process was renewed and soon completed. |
| Case 7128 | 22 yrs. | Soft chancre and suppurating bubo. Bubo was lanced and cauterized, and mercury and iodide of potassium taken twelve days; carbolic acid washes, tincture of iodine, and powdered sulphur were successively used internally, without much benefit. | Chancre very slowly improving; bubo looking a little worse—more excavated perhaps. | Cundurango ordered. On the sixth day the ulcers showed marks of improvement. Bubo touched with nitrate of silver that day. On the tenth day the chancre was nearly healed, and the bubo so far improved that the patient was discharged as convalescent. |
| Case 7137 | 35 yrs. | Suppurating bubo; the chancre (caught four weeks ago) is now healed. Bubo not open. Had taken some unknown medicine. | Lanced the bubo and cauterized the interior. Carbolated water as a wash, and simple cerate dressing. | Cundurango ordered. Cavity began to heal at once, but more rapidly after the fifth day. Discharged on the fourteenth day, nearly cured. |
| Case 7141 | 28 yrs. | Indolent ulcer on the shin, not of large size, but of long standing. Treated eight months by the various usual methods, including skin grafting and afterwards a plastic operation by flaps. Case excessively obstinate and inert. | Ulcer indolent and a little increasing in size. Patient well nourished and reasonably vigorous. No perceptible reason for the indolence of the sore. | Cundurango ordered. Began to improve about the eighth day, and now (30th day) the ulcer is about one-half healed, and still improving. |
| Case 7144 | 9 yrs. | Hip disease in third stage. Discharging pus from several fistulas. Been on the use of tonics and local antiseptic dressings, and injections for ten months. | Has improved very slowly. Has nine ulcers, very sensitive to touch. System somewhat exhausted. | Ordered cundurango, and continue the antiseptic dressings. Appetite increased, bowels move oftener; gains flesh and color; granulations became abundant and the pus laudable. Now, at the thirty-eighth day from commencing the medicine, the general condition is excellent, the granulations almost superabundant, the soreness diminished, and the ulcers greatly reduced in size, but the fistulas not closed. |
| Case 7527 | 48 yrs. | Small indolent ulcer on the cheek, remaining after excision of a cancer. The ulcer itself showed no cancerous character. | The ulcer is flat, small and simply indolent, and not improving. | Ordered cundurango. Third day, granulating more freely; sixth day, granulations increasing; eleventh day, about half healed. |
| Case 7547 | 35 yrs. | Ulceration of cervix uteri and painful micturition. | Cervix swollen, and has an ulcer about an inch in diameter. Stomach irritable; bowels rather constipated. Frequent and painful micturition. | Six days before taking cundurango, cauterized with nitrate of silver, and prescribed astringent injections, with carbolated oil applied to the cervix on cotton; also tannate of quinia internally. Patient felt somewhat better. Changed to cundurango; continue local treatment. Tenth day after change to cundurango, the ulcer was about half healed; dysueria gone; constipation remedied; general condition improved. Fourteenth day, ulcer still further healing. |

CUNDURANGO HAS NO EFFECT ON HARD CHANCRE.

The experiments on this point are not as numerous as is desirable, but so far as they go seem to show that neither hard chancre nor the earlier secondary eruptions are influenced by the medicine. The early stages of secondary syphilis are marked by a hyperplastic diathesis. The eruptions on the skin are of a dry and scaly character, and are the very opposite of the ulcerative pustules of the tertiary stage. Hence the capacity of the drug to produce granulations finds no ulcerative field on which to work. Yet in one case there existed a small abscess, accompanying the scaly plastic eruption, and in this instance, though the cundurango had no effect on the eruption, it produced an exuberant growth of granulations on the seat of the abscess (case 7958). I infer from this that, though early secondary eruptions are little affected by the drug, perhaps the ulcerations of the same stage might fare better.

The following cases contain all my experiments on hard chancre and early secondary syphilis:

Case 7135.—Exposed to contagion six weeks ago, from which he took hard chancre. Has had no treatment of any kind, and now presents himself with the chancre still existing, and a characteristic coppery eruption just commencing on his forehead. Ordered cundurango internally, and a simple astringent wash to the chancre. He took the drug twelve days, without in the slightest degree arresting the progress of the disease or modifying the chancre. I now abandoned the cundurango, and administered mercurials. In ten days after the change the eruption was disappearing, and the chancre healing. In seventeen days both troubles were so nearly cured that the patient was discharged. As he took the remedy only twelve days, it is not certain but he might have derived benefit from a more persevering use of it; but still in ulcers the effect is usually visible in less than ten days, and the tendency of the case is toward the idea that the drug has no effect on constitutional syphilis in the early stages.

Case 7958.—Contracted a small hard chancre six months ago, which healed on being sprinkled with calomel. He now presents himself with a copious eruption of dry, coppery papulæ with flattened, scaly summits. There is also a small abscess in the groin, and slight ulcerations of the throat. Lanced the abscess, and prescribed iod. potass. At the end of thirteen days no effect had been obtained, and cundurango was given with the other medicines. Ten days of this treatment produced no effect on the eruption, but there was a great exuberance of granulations in the seat of the abscess. The cundurango was now omitted, and the mercurials and iodide continued alone, which up to the present time have produced no effect.

In this case, it is evident that the cundurango had a positive effect on the ulcer l[illegible] by the abscess, but none on the dry, plastic eruption. As in the previous case, the experiment was not continued long enough to be decisive. It is to be noticed, also, that the eruption equally resisted the mercurial.

Case 7550.—A week after exposure to contagion this patient presents himself, with three small chancres, which at first were soft, but subsequently two of them became indurated. Cauterized with nit. acid, and ordered cundurango internally and zinc wash locally. Third day, no improvement; fourth day, no improvement; seventh day, no improvement; eleventh day, begins to heal slowly. There is no apparent effect, therefore, on the ulcer. It is not yet possible to determine whether the drug will, in his case, prevent secondary manifestations.

These three cases of hard chancre and early secondary eruption are not enough to settle decisively the effect of cundurango in this stage of syphilis; yet they rather point to the conclusion that it has no effect on the primary *hard chancre*, nor on the *early, dry eruptions*, but nevertheless benefits secondary ulcers as it does the tertiary ones.

Tertiary syphilis consists essentially in the deposit in the tissues of that cacoplastic effusion which has a tendency to break down by ulceration, and whose feeble abscesses are often called gummy deposits. This deposit is chiefly found months or years after the contagion, and from its late appearance its effects are called tertiary; yet there is no clear line of distinction between the secondary and tertiary stages, and it is probable that in many cases deposits identical with the tertiary commence to form during what is commonly called the secondary stage. If so, the remedy might cure many ulcers and eruptions in the secondary stage, though its chief usefulness were in the tertiary.

### EXPERIMENTS ON CANCER.

I have been unable to produce the least effect with cundurango on the size and progress of cancerous tumors, although I have observed in some patients a great diminution of pain, a correction of the offensiveness of the discharge, and a magnificent tonic influence on the general system. Table II. shows the details of the cases, some of which were under my own care, and others were reported to me by reliable Chicago physicians.

From this table it will be noticed that in no instance have I seen any effect of the drug upon the growth and progress of a cancerous tumor, although I have generally observed a relief from pain, and often a remarkable improvement in the general health. Where the cancer had been removed with the knife, the cundurango exerted its usual powerful influence in the healing of the wound, as, for example, in case 6240. In short, cundurango has no power to arrest the cancer, but benefits the patient materially in other respects. I think this will be found to be the almost universal opinion of those who have tested its effects, both in this country and Europe.

Of exceptions to this rule I have found not a single one, but the following three cases have been communicated to me by letter from persons whom I do not personally know. The first two were given me in a private letter by Dr. H. Chiribaga, Surgeon-in-Chief of the Army of Ecuador.

Case 1.—Cancer of the tongue. Cured by cundurango without operation.

Case 2.—Cancer of the right breast; not ulcerated. Under the use of cundurango it disappeared by resolution.

The remaining case occurred in Buffalo, and is given by another writer as follows:

Case 3.—Cancer of the breast. Removed three times by operation, and always returned. At the last return it attained a diameter of three inches, and was quite hard. Cundurango was then commenced, under the influence of which the tumor is said to have become resolved, and to have disappeared in six weeks.

Never having seen these patients, I am unable to say whether the diagnosis was correct, or whether the progress of the cases is correctly stated. Dr. Chiribaga, of Ecuador, however, sustains such a high and honorable character that his observations must have great weight; yet if the patients he saw were not under his own daily care, he might have been mistaken about the diagnosis. At any rate, I have been unable to find any cases of cancer cured by cundurango in or around Chicago.

The conclusions to which our experiments lead seem obviously to be as follows:

1. Cundurango has no equal in the materia medica in its power to increase the growth of granulations, and hasten the cicatrization of ulcers.

2. As a corollary of this proposition, it seems probable that not only external ulcers, but chronic abscesses and some fistulas, may be benefited by it. It may improve lumbar abscesses, for instance.

3. It probably may prove a valuable adjuvant to the treatment of ulcers of the nasal passages, of ulcerative cavities in the lungs, of ulcers of the mucous membrane, of the bowels, of the cervix uteri, etc., and possibly of all ulcerative and pustular skin diseases.

4. The growth of granulation cells is the same process, whether seen on the floor of an ulcer, around a fractured bone in the form of a provisional callus, or in the plastic effusion which glues together the lips of a wound healing by first intention. It is probable, therefore, that cundurango, given as preparatory treatment, may greatly favor the success of cutting operations; and administered after surgical accidents, may promote the union of fractured bones, and the healing of open wounds and burns.

5. Cundurango acts favoraby in soft chancre (after cautery), and on the ulcers and eruptions of tertiary syphilis.

6. My experiments on hard chancre are not yet extensive enough to be decisive, but, as far as they go, they tend to the idea that cundurango has no influence on hard chancre, nor on the earlier eruptions of secondary syphilis.

7. The remedy has not produced, under my observation, any effect on the growth and progress of cancerous tumors. It however often diminishes the pain and the discharges, and, if the tumor has been excised, hastens the healing of the wound.

8. Cundurango generally increases the appetite, diminishes constipation, and acts on many patients as a very powerful general tonic.

No. 6 Sixteenth street, Chicago, Ill.

### TABLE II.

### CASES OF CANCER TREATED BY CUNDURANGO.

| NUMBER OF THE CASE IN MY RECORD. | DIAGNOSIS AND HISTORY. | CONDITION AT THE TIME OF COMMENCING THE MEDICINE. | SYMPTOMS AND PROGRESS WHILE TAKING THE REMEDY. |
|---|---|---|---|
| Case 6713........ | Soft cancer of the thigh. | Tumor is just below Poupart's ligament, four inches in diameter; not yet open; not much cachexy present. | Took internally a mixture of fluid extract cundurango, Fowler's solution and carbolic acid. A florid rash appeared on the skin with a prickly sensation Free sweating for two hours after each dose. Tumor not benefited. It soon opened, and patient died in about sixty days. |
| Case 7482........ | Scirrhus of left breast of nine years' standing. Commenced ulcerating two years ago. | Tumor hard; shooting pains well marked; ulceration indolent; no perceptible cachexy. | Cundurango externally and internally. The shooting pains ceased and changed to a steady aching and tenderness. Discharge increased in quantity and more like healthy pus. She at first thought the tumor decreased in prominence, but after taking the remedy over two months, could perceive no essential change. |
| Professor Byford's Patient. | Cancer of the cervix uteri. | Ulcerated. | Took cundurango about three months with no effect on the tumor. The medicine somewhat irritated the stomach. |
| Professor Byford's Patient. | Soft cancer of the uterus. | Scarcely at all ulcerated. | Took cundurango a number of weeks with no perceptible effect. |
| Case 6232........ | Scirrhus of the rectum of two years' standing. | Tumor ulcerated, tender, painful; discharges foul. | Ordered cundurango. Second day after felt better as to pain. Seventh day, continues to improve in feelings; discharge abated and less offensive. Tenth day, more pain; twelfth day, easier again; twentieth day, examination showed the tumor unchanged, patient weak, but the pain is much diminished; there is also more appetite and better color in the face; twenty-sixth day, appetite better, tumor unchanged; twenty-eighth day, increased pain and discharge; thirty-fourth day, ditto; seventieth day, no better, discouraged, and stopped the medicine. Six months later the tumor was somewhat increased in size. Ordered cundurango, galium aparine, and arsenite of potash in mixture. Nine days after felt relieved; twenty-one days, the tumor has closed the anus, rendering an operation necessary to relieve the intestines. Five months later still, the tumor extending up to the rectum. Has taken no cundurango for some months; gradually wearing out. Ordered a mixture of cundurango, galium aparine and Fowler's solution. The ninth day after was improved in his feelings, but the growth of the tumor was not arrested then nor at any subsequent time. |

TABLE II.—CONTINUED.

| NUMBER OF THE CASE IN MY RECORD. | DIAGNOSIS AND HISTORY. | CONDITION AT THE TIME OF COMMENCING THE MEDICINE. | SYMPTOMS AND PROGRESS WHILE TAKING THE REMEDY. |
|---|---|---|---|
| Case 6239 | Had scirrhus of right breast seven years. | Tumor indolent and slowly ulcerating. | Ordered cundurango. Sixth day, pain diminished; sixteenth day, has continued to feel more comfortable until to-day, when there is increase of pain again; twenty-first day, easier again; twenty-eighth day, still more free from pain; tumor not perceptibly changed. |
| Case 6240 | Soft cancer of the breast. This case was subject to sloughing inflammation on slight causes. Once the insertion of a lancet caused the whole mass to slough out, but the growth returned. | Patient took a journey by railroad to a distant city, to be treated by cundurango. The jarring of the cars (probably) caused a renewed sloughing, with hemorrhage. The surgeon therefore excised the tumor. | Commenced cundurango sometime before the operation, and continued it eight months. The wound commenced healing with remarkable facility and vigor, as I am informed, and the patient felt better than for years; fifteenth month, there remains a very small unhealed spot, but no appearance of return of the cancer. General health greatly benefited. |
| Case 7557 | Tumor of thirty years' standing. Been removed three times, and always recurred. Tender and has some shooting pains. It is very doubtful whether this is really a cancer; it may be a fibrous tumor. | General health moderate; some dyspepsia; tumor painful and somewhat tender. | Commenced cundurango and took it fifteen months in double doses. Dyspepsia was cured; general health became very vigorous and all pain and tenderness left the tumor, but its gradual enlargement has not been arrested, and it is still slowly growing. The remedy has affected this patient as a magnificent general tonic. |
| Professor Powell's Patient. | Soft cancer in the neck. | Not stated. | Commenced taking infusion of cundurango prepared according to directions in circular accompanying the medicine. It was continued four months without diminishing the pain or checking the growth of the tumor. Patient has since died of the disease. The article used was obtained from Washington. |

EXCISION OF INFERIOR MAXILLA.—J. Lizars Lizars, M. D., of Toronto, Canada (*Canada Lancet*, Oct., 1872), describes a case of excision of the inferior maxilla for an enlargement of this bone, extending from about one inch below the zygoma to a line perpendicular from the angle of the mouth, in a man aged 37 years, which was performed as follows: One straight incision was made from the angle of the mouth towards the upper part of the lobe of the ear, as far as the posterior margin of the ascending ramus of the maxilla, the periosteum of the jaw, the masseter, and that part of the temporal muscle attached to the outer and lower part of the coronoid process being denuded by the handle of the scalpel and fingers. The internal maxillary artery was avoided. The proximal end of the facial and inferior dental were the only arteries requiring ligature, a few smaller branches being arrested by torsion. In this case paralysis and salivary fistula were completely prevented.

In speaking of the modes of operation, he inquires which is preferable, a simple scar across the cheek and full power of all the muscles of expression, or a scar which must show more or less a staring eye, a mouth dragged to one side and devoid of play, with probably a constant dribbling of saliva from one corner of it? He most firmly believes that every man or woman of ordinary sense would prefer the former.—*Medical Record.*

CHLOROFORM ADMINISTERED DURING SLEEP.—It has been denied by some prominent scientists, that chloroform can be successfully administered to persons sleeping for the purpose of robbery. We have never doubted the possibility. A case in point is stated in the London *Lancet*, though robbery was not the purpose. Dr. Whitmarsh was about to circumcise a very nervous boy of six years, and finding him asleep, he caused him to inhale chloroform in ten drop doses, until he was completely anæsthetized. The operation was then performed, and the boy waked half an hour afterwards. Dr. Whitmarsh suggests that advantage might be taken of this procedure in nervous persons, who, when awake, often require large, and possibly dangerous quantities of the anæsthetic to overcome the mental tension.—*Pacific Med. Jour.*

